# Near-optimal Individualized Treatment Recommendations

**Published:** 2020

**Authors:** Haomiao Meng, Ying-Qi Zhao, Haoda Fu, Xingye Qiao

**Affiliations:** Department of Mathematical Sciences, Binghamton University, State University of New York, Binghamton, NY 13902, USA; Public Health Sciences Division, Fred Hutchinson Cancer Research Center, Seattle, WA 98109, USA; Eli Lilly and Company, Indianapolis, IN 46285, USA; Department of Mathematical Sciences, Binghamton University, State University of New York, Binghamton, NY 13902, USA

**Keywords:** individualized treatment recommendation, set-valued classification, angle-based classification, reproducing kernel Hilbert space, statistical learning theory

## Abstract

The individualized treatment recommendation (ITR) is an important analytic framework for precision medicine. The goal of ITR is to assign the best treatments to patients based on their individual characteristics. From the machine learning perspective, the solution to the ITR problem can be formulated as a weighted classification problem to maximize the mean benefit from the recommended treatments given patients’ characteristics. Several ITR methods have been proposed in both the binary setting and the multicategory setting. In practice, one may prefer a more flexible recommendation that includes multiple treatment options. This motivates us to develop methods to obtain a set of near-optimal individualized treatment recommendations alternative to each other, called alternative individualized treatment recommendations (A-ITR). We propose two methods to estimate the optimal A-ITR within the outcome weighted learning (OWL) framework. Simulation studies and a real data analysis for Type 2 diabetic patients with injectable antidiabetic treatments are conducted to show the usefulness of the proposed A-ITR framework. We also show the consistency of these methods and obtain an upper bound for the risk between the theoretically optimal recommendation and the estimated one. An R package aitr has been developed, found at https://github.com/menghaomiao/aitr.

## 1. Introduction

The individualized treatment recommendation (ITR) has drawn increasing attention in recent years. Because patients may respond differently to the same treatment (Lesko, 2007; Insel, 2009), it is desirable to individualize the treatment according to patients’ characteristics. Mathematically, an ITR is a map from such characteristics (the covariates, or features) to a treatment. The goal is to find the optimal treatment so that the average benefit that patients will receive by following such a recommendation is maximized.

In the literature, many statistical approaches have been proposed for solving the ITR problem. In indirect modeling-based methods, one first builds a parametric or semiparametric model to estimate the expected outcome conditional on the covariates, then recommends the single treatment that renders the optimal outcome to the given patient (Robins, 2004; Qian and Murphy, 2011; Schulte et al., 2014). However, these methods require a correct model specification and an accurate estimation to perform well in practice. One may also obtain the optimal ITR directly. Zhao et al. (2012) proposed a classification-based method, coined as the outcome weighted learning (OWL), to estimate the optimal ITR. They transformed the ITR problem into a weighted classification problem and used support vector machine (SVM), a classification method, to solve it. Built on top of the OWL framework, there has been a rapidly growing literature on different aspects of the ITR problem. Zhao et al. (2014) and Cui et al. (2017) extended the OWL framework to accommodate survival outcomes. Zhou et al. (2017) and Liu et al. (2018) proposed residual weighted learning (RWL) and augmented outcome-weighted learning (AOL) respectively to reduce the variability of the weights in OWL to enhance its performance. Chen et al. (2018) proposed generalized OWL (GOWL) to solve an ITR with ordinal treatments. Zhang et al. (2018a) proposed angle-based approach for the multicategory case (in which there are more than two treatments available to choose from). Recently, Zhao et al. (2019) and Huang et al. (2019) considered replacing the weights in OWL with a doubly robust estimator to further improve the robustness of OWL. Methods based on other learning algorithms such as trees (Laber and Zhao, 2015; Kallus, 2016; Doubleday et al., 2018; Zhu et al., 2017) and nearest neighbors (Zhou and Kosorok, 2017; Wu et al., 2019) have also been studied. Another example of direct methods is the work by Zhang et al. (2012), which searched for the ITR among a pre-specified class of decision rules that optimized a doubly robust augmented inverse probability weighted estimator of the overall population mean outcome.

Despite the success of these methods in recommending a *single* “optimal” treatment to patients, a method that can suggest multiple “near-optimal” treatment options to a patient is not fully studied. Such options could be desirable when several treatments have comparable effects. Laber et al. (2014) and Lizotte and Laber (2016) proposed a set-valued dynamic treatment regime. In particular, if there are two treatments available (1 and −1), their set-valued rule may report {1}, {−1}, or {1, −1}. However, this approach is applicable only to cases with two competing outcomes. They would recommend the set {1, −1} if any treatment cannot be proven to be inferior to the other based on both outcomes. On the other hand, they used a regression-based method to estimate the optimal set-valued rule, which may suffer an inconsistency issue if the model is misspecified. Yuan (2015) considered a framework to allow a reject option in ITR estimation based on OWL. However, the method is restricted to the binary case with only two possible treatments.

In this paper, we propose to study the ITR problem in the setting with only one clinical outcome from a new perspective. Different from the previous ITR work, it provides a set of treatment options that are near the optimality and are alternative to each other, which we called alternative individualized treatment recommendations (A-ITR). Specifically, if multiple treatments are expected to result in similar and near-optimal clinical outcomes for the patient, then they are all recommended to the patient (or the physician), who can choose any one of them to use after incorporating other considerations. There are several reasons this approach may be more desirable than a single treatment recommendation. First, since multiple treatments may yield the same or similar outcomes for some patients, the ranking among the near-optimal treatment options may vary randomly. If only the top treatment is reported, such a seemingly “random” recommendation may severely undermine the trust of the physicians and the patients toward the treatment recommendation system. Second, when the expected outcomes for multiple treatments are indistinguishable, it may be both legally and morally inappropriate to withhold such important information from the patients. Third, A-ITRs allow physicians and patients to incorporate other factors into their decision-making process regarding the treatment. These other factors include the healthcare expense, the painfulness of the treatment, the side-effect of the treatment, the life quality and lifestyle, and so on. For example, when two treatments are expected to have similar outcomes, it is reasonable to choose an option that is covered by the insurance, that is less painful, that has less side-effect, and that does not significantly compromise the quality of life. In this sense, conventional ITR methods that only recommend one treatment may prevent patients from making informed decisions about their lives.

We will propose two methods to estimate A-ITR. Parallel to the development of the conventional ITR methods, we first introduce a regression-based plug-in method to estimate the optimal A-ITR, which will serve as the baseline. Within the OWL framework, we propose two classification-based methods. The technical tool we will use is multicategory classification with reject and refine options (Zhang et al., 2018b).

The rest of the paper is organized as follows. In Section 2, we review the background of the ITR and the classification with reject and refine options problems. We then introduce the proposed A-ITR framework and discuss several estimation methods in Section 3. Detailed algorithm and the tuning procedure can be found in Section 4. We demonstrate the empirical performance through simulation studies in Section 5 and a real-world application using Type 2 diabetes mellitus data in Section 6. Theoretical studies of the proposed method can be found in Section 7. Some concluding remarks are given in Section 8. All technical proofs are provided in the supplementary materials.

## 2. Background

In this section, we briefly review the background information of both the ITR problem and the problem of classification with reject and refine options.

### 2.1 Individualized Treatment Recommendation

Denote the covariates of a patient by X∈X. Each treatment is denoted by a random variable *A*, where A∈A={1,2,…,k} (*k* treatments available.) After assigning treatment to a patient, we observe an outcome $Y\in R^{+}$. Here we assume *Y* is bounded. Unlike many other ITR methods, we assume *smaller Y* is preferred due to a small technicality that can allow some computational savings. An individualized treatment recommendation, previously often referred to as an individualized treatment rule, is a map d:X→A.

Let Y*(*j*) denote the potential outcome that would have been observed when treatment *j* is assigned to the patient with covariates ***X***. The actual observed outcome *Y* is related to the potential outcomes by $Y=\sum_{j\in A}Y^{*}(j)1[A=j]$. Define *p*(*A* = *j* ∣ ***X***) as the conditional probability of treatment *j* given ***X***. We assume the following assumption.

**Assumption 1** For any j, Y*(*j*) *is independent of A given* ***X*** *and p*(*A* = *j* ∣ ***X***) > 0 *almost everywhere*.

Under Assumption 1, it was shown by Qian and Murphy (2011) and Kallus (2016) that the expected outcome under ITR *d* is

(1) $$E^{d}(Y)=E(Y^{*}(d(X)))=E[E(Y^{*}(A)|A=d(X),X))]=E\left[\frac{1[A=d(X)]}{p(A|X)}Y\right],$$

where $E^{d}$ is the expectation under ITR *d*. Note that *p*(*A*∣***X***) is usually known in a randomized trial, while in an observational study it is unknown and needs to be estimated.

Denote $\mu_{j}=E(Y\;|\;X,A=j)$, for *j* = 1,…, *k*. Then the optimal ITR d* is

(2) $$d^{*}=\underset{d}{\text{argmin}}E^{d}(Y)=\underset{j}{\text{argmin}}\mu_{j},$$

that is, the optimal treatment for a patient has the smallest (the best) expected outcome.

Many methods have been proposed for estimating the optimal ITR. One method is often called “regression and comparison” or Q-learning (Robins, 2004; Qian and Murphy, 2011). One first estimates the conditional mean $\mu_{j}(x)=E(Y\;|\;X=x,A=j)$ for each treatment *j*, then the optimal treatment is obtained by plugging the estimators in (2). However, this method relies on the accuracy of the regression model. If the model is mis-specified, the error could be fairly substantial. Another group of methods treat the problem as a classification problem. One example is called outcome weighted learning (OWL) or O-learning (Zhao et al., 2012, 2014, 2019; Zhou et al., 2017; Zhang et al., 2018a). In the OWL framework, by noting (1), we rewrite the ITR solution as

(3) $$d^{*}=\underset{d}{\text{argmin}}E\left[\frac{Y}{p(A|X)}1[A=d(X)]\right],$$

which is closely related to a weighted classification problem with weight *Y*/*p*(*A*∣***X***). To overcome the non-continuity and non-convexity of the 0-1 loss, we can replace 1[A=d(X)] by a convex surrogate loss *L*(*A*, ***f***(***X***)) in the empirical counterpart of (3) and solve instead

(4) $$\hat{f}=\underset{f}{\text{argmin}}E_{n}\left[\frac{Y}{p(A|X)}L(A,f(X))\right],$$

where $E_{n}$ denotes the empirical expectation, and ***f*** is a vector-valued function defined on X. The estimated ITR $\hat{d}$ is then obtained from $\hat{f}$.

The relationship between $\hat{d}$ and $\hat{f}$ depends on the choice of the loss function *L* and how ***f*** is defined. Zhao et al. (2012) proposed to replace the 0-1 loss by hinge loss in the binary case (*k* = 2, *A* ∈ {1, −1}), that is, *L*(*A*, *f*) = (1 − *Af*)_+_, where *x*_+_ = max(*x*, 0), and *f* is a scalar-valued function. In the current setting that a smaller *Y* is preferred, they could have used *L*(*A*, *f*) = (1 + *Af*)_+_. They showed that the optimal ITR can be estimated by $\hat{d}=\text{sign}\;(\hat{f})$. Zhang et al. (2018a) then extended to the multicategory case using a large-margin loss under the angle-based learning framework (Zhang and Liu, 2014). Specifically, define $f(x)=(f_{1},\ldots ,f_{k-1}{)}^{T}(x)\in R^{k-1}$ and ***W***_1_, …, ***W**_k_* are vertices of a (*k* − 1)-dimensional simplex with equal pair-wise distances, defined as

Wj={(k−1)−1∕21k−1,j=1−(1−k1∕2)(k−1)−3∕21k−1+[k∕(k−1)]1∕2ej−1,2≤j≤k,}

where **1**_*k*−1_ is a (*k*−1)-dimensional vector with all 1 and $e_{j-1}\in R^{k-1}$ is a vector with the (*j*−1)th element 1 and 0 elsewhere. They let *L*(*A*, ***f***(***x***)) = *ℓ*(⟨***W**_A_*, ***f***(***x***)⟩), where *ℓ* is a typical large-margin surrogate loss for binary classification (except that it is increasing instead of decreasing), and ⟨***W**_A_*, ***f***(***x***)⟩ denotes the inner product of the vectors ***W**_A_* and ***f***(***x***). From the geometry point of view, treatment *j* is represented by vertex *j* of the simplex, and the angle between ***f***(***x***) and ***W**_j_*, ∠(***W**_j_*, ***f***(***x***)), indicates how far away ***f***(***x***) is from each of these treatments. The resulting ITR was estimated by $\hat{d}(x)={\text{argmin}}_{j}∠(W_{j},\hat{f}(x))={\text{argmax}}_{j}\langle W_{j},\hat{f}(x)\rangle$, that is, the best treatment is the one whose corresponding vertex is closest to $\hat{f}(x)$ in terms of the angle.

**Remark 2** In the ITR literature, one typically assumes that larger values of the outcome Y are preferred, so that instead of minimization, d* maximizes the objective (3), or equivalently, ${\text{argmin}}_{d}\;E\left[\frac{Y}{p(A|X)}1[A\neq d(X)]\right]$, *which was indeed a weighted classification problem. In this article, recall that we assume smaller values of Y are preferred. As a consequence,* 1[A≠d(X)] *is replaced by* 1[A=d(X)] *in* (3)*; additionally, the surrogate loss function is flipped with respect to the origin so that it is an increasing function instead of a decreasing function*.

### 2.2 Classification with Reject and Refine Options

We aim to provide set-valued recommendations that are near the optimality and are alternative to each other. To this end, we borrow the idea of multicategory classification with reject and refine options as a technical tool. Classification with a reject option has been widely studied. Herbei and Wegkamp (2006) formulated the problem as a minimization problem under the 0-*d*-1 loss. That is, the loss of a misclassified instance is 1 and the loss of a rejected instance is *d*, where 0 ≤ *d* ≤ 1/2. Bartlett and Wegkamp (2008) proposed an estimation procedure under the hinge loss. Yuan and Wegkamp (2010) extended this framework to a broad class of surrogate loss functions. Zhang et al. (2018b) generalized it to the multicategory case.

We first introduce binary classification with reject option. Let (***X***, *A*) be a pair of random variable with X∈X and class label *A* ∈ {1, −1}*, and denote *p_j_*(***x***) = *p*(*A* = *j* ∣ ***X*** = ***x***) as the conditional class probability given ***X***. The goal is to train a classifier *ϕ*(***x***) that produces three possible outputs: 1, −1, and 0. Here 0 stands for a “reject” option, meaning that the classifier refuses to make a prediction based on the information available. Note that the decision “0” can be viewed as a set-valued decision of {1, −1}. Chow (1970) proposed the 0-*d*_0_-1 loss with corresponding risk function *P*(*ϕ*(***X***) ≠ *A*, *ϕ*(***X***) ≠ 0) + *d*_0_*P*(*ϕ*(***X***) = 0) and it was shown that the Bayes rule under this risk is

ϕ∗(x)={1,p1(x)>1−d0−1,p1(x)<d00,d0≤p1(x)≤1−d0.}

Here *d*_0_ ∈ [0, 1/2] controls the cost for refusing to make a classification. Intuitively, we produce the reject option “0” only when both *p*_1_(***x***) and *p*_2_(***x***) are close to 1/2. Bartlett and Wegkamp (2008) proposed a bent hinge loss to estimate the optimal rule ϕ*. The bent hinge loss is defined as *ℓ*(*u*) = max(0, 1 − *u*, 1 − (1 − *d*_0_)*u*/*d*_0_), i.e., the common hinge loss with a bent slope at 0. The effect of such bent slope is to shrink *f*(***x***) to 0 when *p*_1_(***x***) and *p*_2_(***x***) are close. For $f^{*}(x)={\text{argmin}}_{f(x)}\;E(\ell (Af)\;|\;X=x)$, we have ϕ* = sign(f*).

The situation is much more complicated for multicategory classification. Suppose there are 3 classes, that is, A={1,2,3}, then the possible values for the classifier *ϕ*(***x***) are {1}, {2}, {3}, {1, 2}, {1, 3}, {2, 3}, and {1, 2, 3}. In general, assuming there are *k* classes, *ϕ*(***x***) can be any element in the power set of {1,…, *k*} (except the empty set). In addition to the reject option, which can be understood as the full set {1,…, *k*}, Zhang et al. (2018b) introduced the so-called refine option, which is a set-valued decision with cardinality strictly greater than 1 and less than *k*. It contains all those class labels which are nearly as plausible as the most plausible class. Zhang et al. (2018b) proposed to use a class of loss functions in conjunction with the angle-based learning framework (Zhang and Liu, 2014) to train a set-valued classifier that can render these different options. We note that both the reject option and the refine option are set-valued decisions, and they are analogous to the set-valued recommendations in this work.

## 3. Methodology

In this section, we introduce the framework of alternative individualized treatment recommendations (A-ITR) and propose two methods to estimate the optimal A-ITR.

### 3.1 A-ITR Framework

There are several situations in which ITRs with additional alternative options are desirable. Even with small errors, when several treatments are near the optimality, the ranking of these treatments based on their estimated outcomes may differ from their true ranking. In this case, reporting only one treatment based on the estimated value is problematic. Secondly, when the error in the learning problem is substantially large, the so-called optimal treatment reported by conventional ITRs may lead to an outcome that is much worse than some of the other treatment options. In these situations, recommending a single treatment only adds to the distrust that patients may already have towards such black-box algorithms that they know little about. On the other hand, A-ITR provides a safety net, preventing from committing to a single treatment that is only one out of multiple treatments with similar or indistinguishable outcomes. Morally, as patients are more mindful about their financial responsibility and their quality of life, it is more appropriate to present these alternative options and have the patients themselves to make an informed decision, especially when many of these decisions are life-changing.

An A-ITR is a set-valued map $\phi \;:X\to 2^{A}\setminus \emptyset$. Inspired by the idea of classification with reject and refine options, given a user-predefined number *c* ≥ 1 that defines the scope of the near-optimal treatments, we formally define the optimal A-ITR as,

(5) $$\phi^{*}(x)=\{j\;|\;\mu_{j}(x)∕\mu_{\left(1\right)}(x)\leq c\},$$

where *μ*_(1)_ is the smallest conditional mean outcomes. This optimal A-ITR set contains all the treatment options with *μ_j_* close enough to that of the optimal one, up to a multiplicative constant *c*. Recall that we assume smaller *Y* is preferred. Note that for certain *c* and ***x***, ϕ*(***x***) may contain only one element, that is, the treatment with the smallest mean outcome, which corresponds to the conventional ITR. If it includes all the treatments, it is a non-informative recommendation, analogous to the reject option in set-valued classification problem. Here we call *c* the near-optimality constant.

When *c* = 1, the optimal A-ITR reduces to the optimal ITR defined in (2) for all ***x*** since ϕ* = {*j* ∣ *μ_j_*/*μ*_(1)_ ≤ 1} = {*j* ∣ *μ_j_* = *μ*_(1)_} = argmin_*j*∈{1,…,*k*}_ *μ_j_*. This means that the proposed optimal A-ITR generalizes the conventional optimal ITR.

**Remark 3** *The choice of the near-optimality constant c is made in consultation with the physicians by taking into account meaningful domain knowledge in the clinical context. Note c is not a tuning parameter and is not meant to be selected in a way to minimize some risk or obtain some optimal model (whatever it means). The value of c reflects the physician’s judgment about how close is “indistinguishable” and may vary a lot depending on the application. One value of c (say 10) may be appropriate for one clinical outcome but can be too big for another. In practice, c is chosen according to the physician’s experience, or by empirical data if available. A possible candidate for c is an estimate to* exp[*SD*(log(Y*) ∣ ***x***)]. *Moreover, the physician may also try two or three different c values which may lead to recommendations with varying cardinalities. These recommendations may then be presented to the patient in the order of increasing cardinality. Caution should be exercised when communicating about these new alternative options and the corresponding possible sacrifice to the outcome*.

**Remark 4** The optimal A-ITR ϕ* defined in (5) *is not invariant to addition but is invariant to multiplication. If it were invariant to addition, then the assumption of non-negativity of Y would be moot. In practice, many clinical outcome are positive (i.e., year of survival, blood count, etc.). A negative clinical outcome* $\tilde{Y}$ *(i.e., a decrease of blood pressure) may be transformed to be positive, for example, by* $Y=\text{exp}(\tilde{Y})$. *In any case, a transformation of the original clinical outcome may be needed to adapt the definition* (5) *to the specific clinical context. For example, in certain clinical contexts, it could make more sense to define the optimal treatment options as those with expected outcomes less than* ${\tilde{\mu }}_{\left(1\right)}+b$ *where* ${\tilde{\mu }}_{\left(1\right)}$ *is the smallest mean outcome (in the original, untransformed scale). In this case, we may define* $Y=\text{exp}(\tilde{Y})$ *so that the optimal treatment options are those with expected (transformed) outcome less than* *μ*_(1)_ × *c with c* = exp(*b*). *See* Section 6 *for a real data example in which some data transformation is done*.

### 3.2 Estimation

We consider two types of methods to estimate the optimal A-ITR: the regression-based methods and the classification-based methods. For regression-based methods, we can use Q-learning to first estimate the conditional mean $\mu_{j}(x)=E(Y\;|\;X=x,A=j)$ for each treatment *j*, then plug into (5), i.e., $\hat{\phi }(x)=\{j\;|\;{\hat{\mu }}_{j}(x)∕{\hat{\mu }}_{\left(1\right)}(x)\leq c\}$. The success of this regression-based plug-in method relies on accurate estimation of *μ_j_*.

In contrast, the classification-based method targets on estimating the true boundary between different decision regions, bypassing the need to estimate *μ_j_* directly. In the rest of the section, we propose two classification-based methods within the OWL framework, both of which are based on the angle-based learning approach (Zhang and Liu, 2014).

Zhang et al. (2018a) first made use of the angle-based learning approach to solve the ITR problem, in which they denoted ***W***_1_ …, ***W**_k_* as the vertices of a (k − 1)-dimensional simplex and they chose the loss *L*(*A*, ***f***(***x***)) in (4) to be a function that only depends on the inner product ⟨***W**_A_*, ***f***(***x***), namely, *L*(*A*, ***f***(***x***)) = *ℓ*(⟨***W**_A_*, ***f***(***x***)⟩). Define f* to be the population minimizer under such loss, that is

(6) $$f^{*}=\underset{f\in \{X\to R^{k-1}\}}{\text{argmin}}E\left[\frac{Y}{p(A|X)}\ell (\langle W_{A},f(X)\rangle )\right].$$

The end product of Zhang et al. (2018a) was a single-treatment ITR. In the ideal case that f* can be obtained, their ITR was defined as *d*_f*_(***x***) = argmax_*j*∈{1,…,*k*}_,⟨***W**_j_*, f*(***x***)⟩, and it can be shown that as long as *ℓ* is convex and strictly increasing, Fisher consistency holds, i.e., d_f*_ = d*. In practice, given the training data set $\{(x_{i},a_{i},y_{i}){\}}_{i=1}^{n}$, $\hat{f}$, the estimate of f*, is obtained by,

(7) $$\hat{f}=\underset{f\in F}{\text{argmin}}\frac{1}{n}\sum_{i=1}^{n}\frac{y_{i}}{p(a_{i}|x_{i})}\ell (\langle W_{a_{i}},f(x_{i})\rangle ),\;\text{subject to}\;J(f)\leq s,$$

where $F\subseteq \{f\;|\;X\to R^{k-1}\}$ is a class of functions, and *J*(***f***) is a penalty term to prevent overfitting.

Both our proposed A-ITR methods are derived from the empirical minimizer $\hat{f}$ in (7) with an aim to estimate the population minimizer f* in (6). The difference lies in the loss function *ℓ* they use, and how they convert $\hat{f}$ or f* to the final set-valued recommendations.

#### 3.2.1 Two-step OWL METHOD

For the two-step method, we use a convex, differentiable, and increasing loss function *ℓ_D_*. Given any ***f*** (which may be f* or $\hat{f}$), to obtain the A-ITR, it is instrumental to first order the vertices ***W**_j_*’s, *j* = 1,…, *k*, which represent the *k* treatments, in the manner of *reversed* order statistics, ⟨***W***_(1)_, ***f***⟩ > ⋯ > ⟨***W***_(*k*)_, ***f***⟩. It turns out (see Proposition 5 below) that when ***f*** = f*, the *j*th *reversed* order statistic (i.e., the *j*th largest) ⟨***W***_(*j*)_, ***f***⟩ corresponds to the *j*th order statistic (the *j*th smallest) *μ*_(*j*)_ where *μ*_(1)_ < ⋯ < *μ*_(*k*)_.

The resultant two-step estimator of the optimal A-ITR is then defined as

(8) $$\phi_{f}^{D}(x)=\left\{j\;|\;\frac{\ell_{D}^{\prime }(\langle W_{\left(1\right)},f(x)\rangle )}{\ell_{D}^{\prime }(\langle W_{j},f(x)\rangle )}\leq c\right\}.$$

Here $\ell_{D}^{\prime }$ is the first derivative of *ℓ_D_*, and the superscript “*D*” indicates that ***f*** is the solution based on a *differentiable* loss function. Our estimator is motivated by the following result.

**Proposition 5** (Zhang et al., 2018a) *Let* f* *be the population minimizer in* (6) *in which ℓ is a convex and differentiable function ℓ_D_ with* $\ell_{D}^{\prime }(u)>0$ *for all u*. *For any i* ≠ *j* ∈ {1,…, *k*}, *we have*

$$\frac{\mu_{j}}{\mu_{i}}=\frac{\ell_{D}^{\prime }(\langle W_{i},f^{*}\rangle )}{\ell_{D}^{\prime }(\langle W_{j},f^{*}\rangle )}.$$

Proposition 5 implies the following Fisher-consistent-like result for our proposed A-ITR estimator (8).

**Proposition 6** *Let* f* *be the population minimizer in* (6) *in which ℓ is a convex and differentiable function ℓ_D_ with* $\ell_{D}^{\prime }(u)>0$ *for all u*. *The* $\phi_{f^{*}}^{D}$ *defined in* (8) *based on* f* coincides with the optimal A-ITR ϕ* *in* (5).

This method is a two-step procedure because it first estimates f* using $\hat{f}$, then estimates the ratios of conditional means *μ_j_/μ_i_* using $\ell_{D}^{\prime }(\langle W_{i},\hat{f}\rangle )∕\ell_{D}^{\prime }(\langle W_{j},\hat{f}\rangle )$ which is then plugged into (5) to obtain the A-ITR. Note that it does not estimate each conditional mean individually, but their ratios. The issue remains that if f* is not accurately estimated, then the ratio *μ_j_/μ_i_* cannot be accurately estimated.

#### 3.2.2 One-step OWL Method

**Figure 1:**
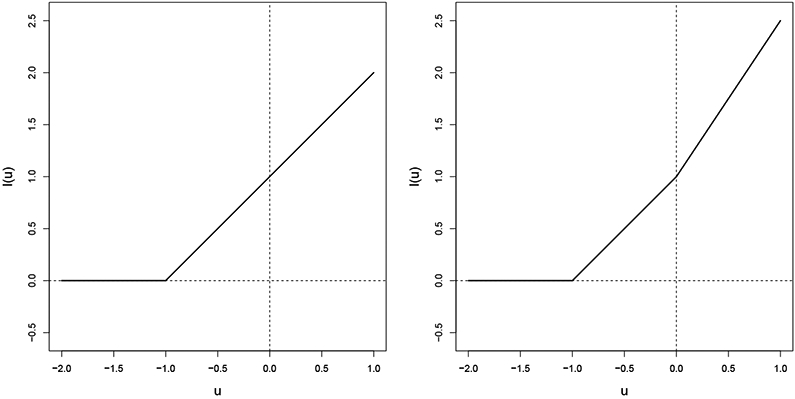
Hinge loss (left panel) and bent hinge loss with *c* = 1.5 (right panel). Note the additional slope at *u* = 0 for the bent hinge loss.

The one-step method aims to directly obtain a set-valued recommendation without calculating $\ell_{D}^{\prime }(\langle W_{i},\hat{f}\rangle )∕\ell_{D}^{\prime }(\langle W_{j},\hat{f}\rangle )$. The crucial difference here is the use of a bent loss function, defined as

$$\ell_{B}(\langle W_{j},f\rangle )=\ell_{1}(\langle W_{j},f\rangle )+\ell_{2}(\langle W_{j},f\rangle ),$$

where *ℓ*_1_ > 0 is a convex and increasing function with $\ell_{1}^{\prime }(u)=1$ for all *u* ≥ 0, and *ℓ*_2_(*u*) = (*c* − 1)*u*_+_ with *c* ≥ 1. Such a loss function is bent at 0, since $\ell_{B}^{\prime }(0-)=1$ and $\ell_{B}^{\prime }(0+)=c$. Note that the slope *c* is the same as the near-optimality constant as defined in (5). An example of bent loss is the bent hinge loss, *ℓ_B_*(*u*) = (1 + *u*)_+_ + (*c* − 1)*u*_+_ (see Figure 1.) The bent loss has been a critical tool that helps to achieve reject (and refine) options in the classification literature (Bartlett and Wegkamp, 2008; Zhang et al., 2018b).

The main effect of the bent loss is to shrink the angle margin for class *j* (or treatment *j* here), defined as ⟨***W**_j_*, ***f***(***x***)⟩, towards 0, similar to the shrinkage effect of the lasso penalty. Likewise, the additional slope *c* − 1 for *u* > 0 is analogous to a penalty parameter in lasso regression, which would encourage a sparse model. Note that here such a shrinkage effect is applied to the classes/treatments with positive angle margins only. Specifically, Proposition 7 below, derived from Proposition 1 in Zhang et al. (2018b), gives the precise values of the angle margins ⟨***W**_j_*, ***f***(***x***)⟩’s with respect to f*, the population minimizer of (6) with the bent loss *ℓ_B_*.

**Proposition 7** *For the sequence μ*_(1)_ < ⋯ < *μ*_(*k*)_, *suppose there exists an integer r* ∈ {1,…, *k* − 1} *such that μ*_(*j*)_/*μ*_(1)_ < *c* *for j* = 1,…, *r and μ*_(*j*)_/*μ*_(1)_ > *c for j* = *r* + 1,…, *k*. *Let* f* *be the population minimizer to* (6) *in which ℓ is a convex and increasing function ℓ_B_ with* $\ell_{B}^{\prime }(0-)=1$ *and* $\ell_{B}^{\prime }(0+)=c\geq 1$. Then we have ⟨***W***_(1)_, f*⟩ > 0, ⟨***W***_(2)_, f*⟩ = ⋯ = ⟨***W***_(*r*)_, f*⟩ = 0, *and* ⟨***W***_(*r*+1)_, f*⟩ = ⋯ ⟨***W***_(*k*)_, f*⟩ < 0. *If such an integer r does not exist, then* ⟨***W***_(*j*)_, f*⟩ = 0 *for j* = 1,…, *k*.

A direct consequence of Proposition 7 is that all the near-optimal treatments (defined as *μ*_(*j*)_/*μ*_(1)_ ≤ *c*) have non-negative angle margins, while the rest have negative angle margins. This naturally leads to the following set-valued recommendation,

(9) $$\phi_{f}^{B}(x)=\{j\;|\;\langle W_{j},f(x)\rangle \geq 0\}.$$

Here ***f*** can be the population minimizer f* (6) or the empirical minimizer $\hat{f}$ (7) and the superscript “*B*” indicates that the loss *ℓ* is a *bent* loss *ℓ_B_*, as opposed to a differentiable loss function in the two-step method.

Proposition 7 implies that $\left\{j\;|\;\mu_{j}(x)∕\mu_{\left(1\right)}(x)<c\}\subseteq \phi_{f^{*}}^{B}(x)\subseteq \{j\;|\;\mu_{j}(x)∕\mu_{\left(1\right)}(x)\leq c\right\}$. The following assumption is necessary to resolve the identifiable issue of (9) and to show its optimality.

**Assumption 8** *For any positive c*_0_, *p*(*μ_j_*(***X***) = *c*_0_*μ_i_*(***X***)) = 0 *for* ∀*i* ≠ *j* ∈ {1,…, *k*} *in which* $\mu_{j}(X)=E(Y\;|\;(X),A=j)$ *is the conditional mean outcome for treatment j*.

Assumption 8 guarantees the two sets, $\left\{x\in X\;|\;\mu_{j}(x)∕\mu_{i}(x)=1\right\}$ and $\left\{x\in X\;|\;\mu_{j}(x)∕\mu_{i}(x)=c\right\}$, where *c* is the near-optimality constant in (5), have measure 0 for any *i* ≠ *j* so that f* is identifiable almost everywhere. Under Assumption 8, we have the following proposition, analogous to Fisher consistency in classification.

**Proposition 9** *Suppose Assumption 8 holds. Let* f* *be the population minimizer in* (6) *in which ℓ is a convex and increasing function ℓ_B_ with* $\ell_{B}^{\prime }(0-)=1$ *and* $\ell_{B}^{\prime }(0+)=c\geq 1$. *The A-ITR* $\phi_{f^{*}}^{B}$ *defined in* (9) *based on* f* coincides with the optimal A-ITR ϕ* (5) *with the near-optimality constant c*.

While Assumption 8 is useful as a technical assumption, it may not hold in certain practical situations. For example, when two treatments have the same conditional mean outcomes for a group of patients, Assumption 8 does not hold for *c* = 1. Another case that it is more likely to fail is when the outcome *Y* can only take finite and discrete values. Even if it does not hold, the proposed methods could still be useful. See Example 3 in Section 5 in which Assumption 8 is violated.

Note that for both classification-based methods, a single-valued ITR can be easily defined by recommending the treatment option with the largest angle margin, that is, argmax_*j*∈{1,…,*k*}_⟨***W**_j_*, ***f***(***x***)⟩.

Unlike the regression-based method, the two classification-based methods do not estimate the conditional mean outcome. The success of the regression-based method relies on an accurate estimation of *μ_j_* at every ***x*** of interest, while reasonable performance is expected for the classification-based methods as long as the estimation is accurate around the “boundaries”. However, the two-step method and the one-step method seem to have different focuses. Both methods start with finding a discriminant function to minimize the outcome-weighted misclassification rate for the purpose of minimizing the expected outcome. As a consequence, both methods have “good” performances near boundaries that distinguish the optimal treatment from the non-optimal treatments for each patient. The one-step method, additionally, uses a bent loss with a shrinkage effect that is capable of determining whether a treatment is *close enough to*, not whether it is *equal to*, the optimal treatment. More precisely speaking, the one-step method calibrates the boundary defined by *μ_j_*(***x***)/*μ*_(1)_(***x***) = *c*. This is theoretically justified by Proposition 7. Hence, the one-step method also has “good” performance near such new notions of boundaries.

**Figure 2:**
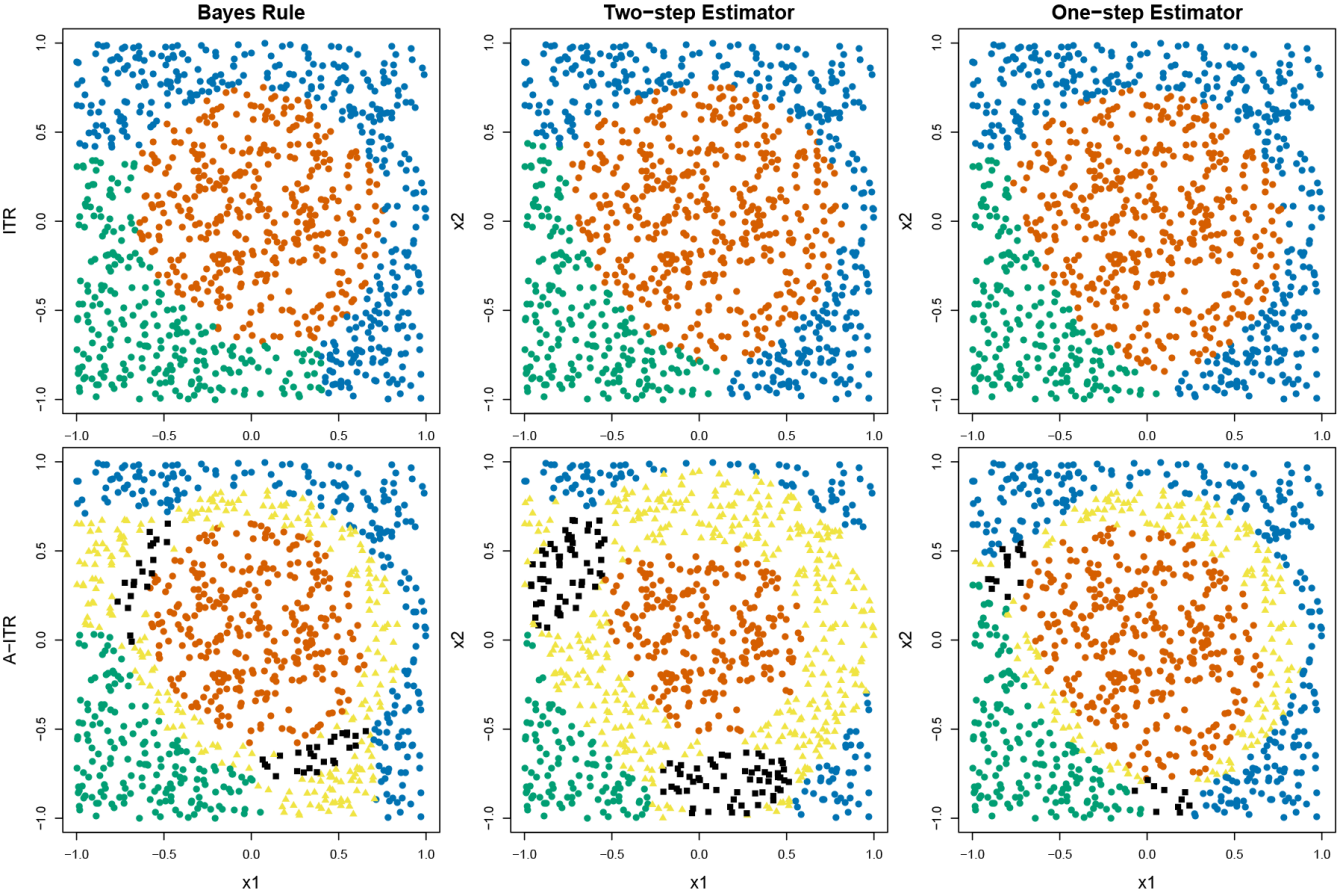
ITR (top) and A-ITR (bottom) for a toy example given by Bayes rule, the two-step estimator, and one-step estimator. Yellow triangles indicate recommendations with two options, and black squares indicate three options. Both estimators give similar results to the Bayes rule in terms of the single-valued ITR. The two-step method does not provide as good A-ITR results as the one-step method.

To illustrate the additional strength of the one-step method, we show the boundaries between recommendations for a toy example (the details of which will be revisited in the numerical studies) in Figure 2, in which the top row shows the single-valued ITR and the second row the set-valued A-ITR, by the Bayes rule, the two-step method, and the one-step method respectively. Both classification-based methods give good approximations to the Bayes ITR boundaries, shown in the top row. However, the two-step method seems to include more treatments into the near-optimal set when compared to the Bayes rule (shown in the bottom row), than the one-step method does. For example, the two-step estimator displays much more recommendations with 2 or 3 treatment options. This is probably due to the fact that the optimization for the two-step method is not designed to capture this subtle pattern, at least not with a finite sample.

## 4. Implementations

In this section, we discuss various aspects of the implementations for the proposed methods, including the optimization, the normalization of the predictive function, and the parameter tuning.

### 4.1 Algorithm

In this section, we introduce the optimization procedure to estimate f* defined in (6). Instead of the constrained problem (7), we solve the regularized problem:

(10) $$\underset{f\in F}{\text{min}}\frac{1}{n}\sum_{i=1}^{n}\frac{y_{i}}{p(a_{i}|x_{i})}\ell (\langle W_{a_{i}},f(x_{i})\rangle )+\lambda J(f),$$

where *λ* is a tuning parameter. It is a weighted classification problem with weight *w_i_* = *y_i_*/*p*(*a_i_*∣***x***_*i*_).

In terms of the function class F, there are linear learning and kernel learning (Steinwart et al., 2007; Hofmann et al., 2008; Hastie et al., 2009). Let $f=(f_{1},\ldots ,f_{k-1}{)}^{T}\in F$, and for simplicity, we add a constant term to ***x***. Then for linear learning, we have *f_j_*(***x***) = ***x***^*T*^*β_j_*, and the corresponding penalty $J(f)=\sum_{j=1}^{k-1}\|\beta_{j}{\|}^{2}=\sum_{j=1}^{k-1}\beta_{j}^{T}\beta_{j}$. For kernel learning, $f_{j}(x)=\sum_{i=1}^{n}K(x_{i},x)\alpha_{ij}+\alpha_{0j}$, where *K*(·, ·) is a kernel function. The penalty term becomes $J(f)=\sum_{j=1}^{k-1}\alpha_{j}^{T}K\alpha_{j}+\sum_{j=1}^{k-1}\alpha_{0j}^{2}$, where **K** is the gram matrix. Note that we include the intercept term into *J*(***f***) and a benefit by doing this is the reduction of the complexity of the algorithm. Zhang et al. (2016) shows theoretically that it can achieve the same convergence rate as the case without the intercept term.

We proposed the two-step method and the one-step method. The two-step method is based on a differentiable loss *ℓ_D_*, while the one-step method is based on a bent loss *ℓ_B_*(*u*) = *ℓ*_1_(*u*) + *ℓ*_2_(*u*), where *ℓ*_1_ is convex and *ℓ*_2_(*u*) = (*c* − 1)*u*_+_. Since *ℓ_D_* is similar to a special case of *ℓ_B_* with *c* = 1, here we only need to focus on the algorithm for the bent loss *ℓ_B_*. In the rest of this section, we use linear learning to demonstrate our algorithm and have deferred the details about kernel learning to the supplementary materials.

We first consider the case when *ℓ*_1_ is differentiable. In this case, we use the ADMM (Boyd et al., 2011) algorithm to solve (10). The ADMM algorithm is used when the objective function can be written as a sum of two convex functions, which, in our case, are *ℓ*_1_ and *ℓ*_2_.

We denote the coefficient matrix as *B*_*p*×(*k*−1)_ = [***β***_1_,…, ***β***_*k*−1_]. Then we create another copy of the coefficients *G*_*p*×(*k*−1)_ = [***γ***_1_,…, ***γ***_*k*−1_], and let *Z*_*p*×(*k*−1)_ = [***z***_1_,…, ***z***_*k*−1_]. Recall that *w_i_* = *y_i_*/*p*(*a_i_*∣***x***_*i*_), then we minimize the augmented Lagrangian

$$L_{\rho }(B,G,Z)=\sum_{i=1}^{n}w_{i}\ell_{1}(\langle W_{a_{i}},B^{T}x_{i}\rangle )+\sum_{i=1}^{n}w_{i}\ell_{2}(\langle W_{a_{i}},G^{T}x_{i}\rangle )+\frac{n\lambda }{2}\sum_{j=1}^{k-1}\beta_{j}^{T}\beta_{j}\;\;+\sum_{j=1}^{k-1}z_{j}^{T}(\beta_{j}-\gamma_{j})+\frac{\rho }{2}\sum_{j=1}^{k-1}(\beta_{j}-\gamma_{j}{)}^{T}(\beta_{j}-\gamma_{j}),$$

where *ρ* > 0 controls the step size.

At step *t*, for each *j* = 1,…, *k* − 1 we can update *B^t^*, *G^t^* and *Z^t^* as

$$\beta_{j}^{t}=\underset{\beta_{j}}{\text{argmin}}L_{\rho }([\beta_{1}^{t},\ldots ,\beta_{j},\ldots ,\beta_{k-1}^{t-1}],G^{t-1},Z^{t-1}),\;\;\gamma_{j}^{t}=\underset{\gamma_{j}}{\text{argmin}}L_{\rho }(B^{t},[\gamma_{1}^{t},\ldots ,\gamma_{j},\ldots ,\gamma_{k-1}^{t-1}],Z^{t-1}),\;\;z_{j}^{t}=z_{j}^{t-1}+\rho (\beta_{j}^{t}-\gamma_{j}^{t})$$

until matrix *B* converges. Note that in the two-step method where *c* = 1, we have *ℓ*_2_(*u*) = 0. In this case, we can force *B* = *G* and only update $\beta_{j}^{t}$’s until they converge.

Next we consider the case when *ℓ*_1_ is not differentiable. In the literature of classification, a non-differentiable loss that has been commonly used is hinge loss. Note that in our case, since we prefer smaller outcomes, we define the hinge loss as *ℓ*_1_(*u*) = (1 + *u*)_+_ (see Figure 1). That is, we flip the traditional hinge loss with respect to the y-axis to make it an increasing function. A typical approach to an optimization problem with the hinge loss is to transform it into a quadratic programming (QP) problem in its duality (Fung and Mangasarian, 2005; Hastie et al., 2009; Zhang et al., 2018b). Specifically, the dual problem of (10) can be written as

$$\underset{\alpha_{j},\gamma_{j}}{\text{min}}\frac{n\lambda }{2}\sum_{j=1}^{k-1}\beta_{j}^{T}\beta_{j}-\sum_{i=1}^{n}\alpha_{i}\;\;\text{s.t.}\;0\leq \alpha_{i}\leq w_{i},\;0\leq \gamma_{i}\leq w_{i},\;i=1,\ldots ,n,$$

where $\beta_{j}=-\frac{1}{n\lambda }\sum_{i=1}^{n}(\alpha_{i}+(c-1)\gamma_{i})W_{ai,j}x_{i}$, and *W*_*a*_*i*_,*j*_ is the *j*th component of ***W***_*a*_*i*__. Note that the weight *w_i_* = *y_i_*/*p*(*a_i_*∣***x***_*i*_) serves as the upper bound of the box constraints. Because the objective function is quadratic in *α_i_* and *γ_i_*, it has explicit solution at each iteration. Thus it converges very fast by using algorithms such as coordinate decent (Zhang et al., 2018b).

In practice, there may be numerical errors to the solution. Moreover, due to different choices of the tuning parameter *λ*, the scale of the resulting angle margins may vary much between different tuning trials. We propose the following normalization procedure for the one-step A-ITR $\phi_{f}^{B}$ (9) to boost the empirical performance. The idea is that instead of recommending all treatments with angle margins greater than or equal to 0, we change the threshold to a small number varying around 0. Such a threshold is a fixed constant *δ* multiplied by a measure of the scale, chosen to be the magnitude of the smallest angle margin. The normalized one-step A-ITR is then

(11) $$\phi_{\hat{f}}^{B}(x)=\{j\;|\;\langle W_{j},\hat{f}(x)\rangle \geq \delta M(x)\},$$

where *δ* is a tuning parameter around 0 and $M(x)=|\langle W_{\left(k\right)},\hat{f}(x)\rangle |$ is the magnitude of the smallest angle margin (note that $\langle W_{\left(k\right)},\hat{f}(x)\rangle$ is negative).

### 4.2 Tuning Procedure

In this paper, the estimation procedure involves two tuning parameters. The first one is the regularization parameter *λ* in (10) which appears in both the two-step and one-step methods. The second one is the normalization parameter *δ* in (11) for the one-step method only. We will tune these two parameters differently in two steps.

The first step is to tune *λ*. For each *λ*, the estimated solution is $\hat{f}$. Then we define the corresponding single-treatment ITR as $d_{\hat{f}}={\text{argmax}}_{j}\langle W_{j},\hat{f}\rangle$ and calculate its empirical average of the expected outcome (1), which is given by

$$\frac{\sum_{i=1}^{n}\left(1\left[a_{i}=d_{\hat{f}}(x_{i})\right]y_{i}∕p(a_{i}|x_{i})\right)}{\sum_{i=1}^{n}\left(1\left[a_{i}=d_{\hat{f}}(x_{i})\right]∕p(a_{i}|x_{i})\right)}$$

(Zhao et al., 2012; Zhang et al., 2018a). We choose the *λ* that yields the smallest empirical risk for the resulting ITR, even if our ultimate goal is to obtain a set-valued A-ITR. This can substantially simplify the tuning process. We found that other more complicated tuning procedures have led to a similar performance.

For the one-step method, we need to continue to tune *δ*. For the same *λ* (same resulting ITR), because different *δ*’s may lead to slightly different set-valued A-ITRs and recommendations with different carnalities, we must actually compare the resulting A-ITRs to choose the best *δ*, instead of using the ITR as a proxy. However, there are some difficulties in evaluating the performance of the estimated A-ITR. Compared to the conventional ITR, the challenge here is that when the recommendation includes two or more treatment options, there are multiple potential outcomes and it is difficult to quantify the “overall” benefit for such a recommendation.

Although the proposed optimal A-ITR ϕ* defined in (5) is not a Bayes rule under any loss function, we can consider a closely related loss function, whose risk function is given by

(12) $$E\left[\frac{Y1[A\in \phi (X)]}{p(A|X)(1+(|\phi (X)|-1)c)}\right],$$

where $\phi \;:X\to 2^{A}\setminus \emptyset$ is a set-valued predictor and ∣ · ∣ denotes the cardinality of a set. Compared to the expected outcome $E^{d}(Y)$ defined in (1), this quantity is a weighted outcome with weight 1/(1 + (∣*ϕ*∣ − 1)*c*) under *ϕ*. If we force ∣*ϕ*∣ ≡ 1, it reduces to $E^{d}(Y)$. More importantly, it can be shown that the minimizer of (12), denoted by *ϕ*^+^, is

$$\phi^{+}(x)=\underset{\phi (x)\in 2^{A}}{\text{argmin}}\mu^{\phi }(x),\;\;\;\text{where}\;\mu^{\phi }(x)≜\frac{1}{1+(|\phi (x)|-1)c}\sum_{j\in \phi (x)}\mu_{j}(x).$$

Here *μ^ϕ^* defines a new criterion that generalizes the expected outcomes under a set-valued treatment recommendation *ϕ*. To see that, note that for *ϕ*(***x***) = {1}, *μ^ϕ^*(***x***) = *μ*_1_(***x***), while for *ϕ*(***x***) = {1, 2}, *μ^ϕ^*(***x***) = (*μ*_1_(***x***) + *μ*_2_(***x***))/(1 + *c*), which is smaller than the simple average (*μ*_1_(***x***) + *μ*_2_(***x***))/2 when *c* > 1. Suppose treatment 1 is better than treatment 2 (*μ*_1_(***x***) < *μ*_2_(***x***)). We can show that *ϕ*_2_(***x***) ≜ {1, 2} is as good as *ϕ*_1_(***x***) ≜ {1} under this new criterion if and only if *μ*_2_(***x***)/*μ*_1_(***x***) ≤ *c*, which is exactly the near-optimal recommendation set defined in (5).

**Figure 3:**
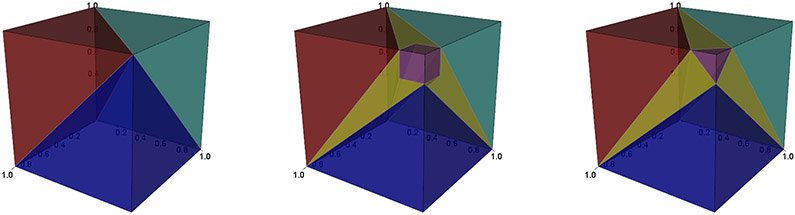
Comparison between ϕ* and *ϕ*^+^ (left: ϕ* or *ϕ*^+^ with *c* = 1; middle: ϕ* with *c* = 1.2; right: *ϕ*^+^ with *c* = 1.2). Any point in the plot represents (*μ*_1_, *μ*_2_, *μ*_3_) (suppose Y*(*j*) ∈ (0, 1)) with the recommendation illustrated by colors. Points in the red, green, and blue regions contain only one treatment; the yellow region contains two treatments; and the purple region includes all three treatments. $S_{1}^{*}=S_{1}^{+}$ (unions of red, blue and green regions), and $S_{2}^{*}\subseteq S_{2}^{+}$ (all but the purple regions).

Intuitively, *ϕ*^+^(***x***) is an optimal set of treatments selected to minimize the “average” clinical outcome with a penalty on the cardinality of the recommendation set. Note that when *c* = 1, *ϕ*^+^ is the same as the optimal ITR d*. Moreover, when *k* = 2, *ϕ*^+^ is the same as the optimal A-ITR ϕ*, as shown above. When *k* ≥ 3, *ϕ*^+^ and ϕ* are different but are nested within each other in the following way: if we let $S_{t}^{*}=\{x\in X\;|\;|\phi^{*}(x)|\leq t\}$ and $S_{t}^{+}=\{x\in X\;|\;|\phi^{+}(x)|\leq t\}$, then we have $S_{1}^{*}=S_{1}^{+}$, and $S_{t}^{*}\subseteq S_{t}^{+}$ for *t* = 2,…, *k* − 1. Figure 3 demonstrates their relationship when *k* = 3.

From Figure 3, we observe that the regions with only one treatment are the same $\left(S_{1}^{*}=S_{1}^{+}\right)$, while the regions containing two or three treatments are slightly different. In general, the boundaries between the size-1 decisions and their complements are the same for the two rules *ϕ*^+^ and ϕ*. They only differ in the boundaries between recommendations with different cardinalities (for example, the boundary between size-2 decisions and size-3 decisions). Although ϕ* does not directly minimize the weighted outcome defined in (12), the similarity between *ϕ*^+^ and ϕ* justifies the use of the weighted outcome (12) as a new criterion for the tuning parameter selection. Specifically, we choose the *δ* value that can yield the smallest value of the following empirical counterpart of (12),

(13) $$\frac{\sum_{i=1}^{n}\left(1\left[a_{i}\in \phi_{\hat{f}}(x_{i})\right]y_{i}∕\left[p(a_{i}|x_{i})(1+(|\phi_{\hat{f}}(x_{i})|-1)c)\right]\right)}{\sum_{i=1}^{n}\left(1\left[a_{i}\in \phi_{\hat{f}}(x_{i})\right]∕\left[p(a_{i}|x_{i})|\phi_{\hat{f}}(x_{i})|\right]\right)}.$$

In addition to the tuning parameter selection, we may also use this criterion to select different methods for conducting A-ITRs. In the real data analysis, we will use this criterion to select between the two proposed classification-based methods.

## 5. Simulation Studies

In this section, we study the numerical performance of the proposed methods.

### 5.1 Comparing Set-valued Recommendations

For two ITRs *d*_1_ and *d*_2_, we can compare them by evaluating the expected outcome defined in (1). However, for two A-ITRs *ϕ*_1_ and *ϕ*_2_, it is difficult to quantify which one is better due to the fact that a measure for the overall benefit is not well defined when multiple treatments are recommended. Although in Section 4.2 we have proposed the weighted expected outcome (12) for evaluating two A-ITRs, the optimal A-ITR *ϕ*^+^ under this new criterion is still different from the desired near-optimal recommendation set ϕ*. So in the simulation studies, in addition to the empirical weighted outcome (13), we consider another means to compare different A-ITRs, using the expected outcome of the best and the worst treatments among the treatments that are recommended, averaged over a set of observations. We conduct such an evaluation for different types of recommendations separately to see how the A-ITR performs differently on them. Based on the size of the true optimal A-ITR ϕ*, we split the covariate space X into three regions corresponding to three kinds of recommendations:

$$R_{1}=\{\text{only one treatment is suggested}\}=\{x\in X\;|\;|\phi^{*}(x)|=1\},\;R_{2}=\{\text{more than one treatment but not all of them are suggested}\}\;\;=\{x\in X\;|\;1<|\phi^{*}(x)|<k\},\;R_{3}=\{\text{all treatments are suggested}\}=\{x\in X\;|\;|\phi^{*}(x)|=k\}.$$

Note that *R*_1_, *R*_2_ and *R*_3_ are disjoint and $X=R_{1}\cup R_{2}\cup R_{3}$. When *c* = 1, ϕ* is the optimal ITR d* and $X=R_{1}$. When *c* > 1, we may have non-empty regions *R*_2_ and *R*_3_.

For two A-ITRs *ϕ*_1_ and *ϕ*_2_, we will compare them separately on *R*_1_, *R*_2_ and *R*_3_. In each region, since multiple treatments may be suggested, we can compare the expected minimal outcome and the expected maximal outcome that they may lead to. Recall Y*(*j*) is the potential outcome by taking treatment *j*. Mathematically, we consider a performance interval,

(E[E((minj∈ϕ(X)Y∗(j)∣X)],E[E((maxj∈ϕ(X)Y∗(j)∣X)]),

where the first quantity indicates the expected outcome if one can always use the best treatment within the recommended set *ϕ*(***x***) and the second quantity represents the worst situation, i.e., how bad it can be if one always chooses the worst treatment among the recommended options. Note that on *R*_1_, the two quantities are the same under ϕ* since only one treatment is recommended. As we increase *c*, we expect that this interval becomes wider on *R*_2_ and *R*_3_ since the diversity of the recommended options increases. From the definition of this interval, we claim that *ϕ*_1_ is better than *ϕ*_2_ if both the lower and the upper limits of this interval under *ϕ*_1_ are smaller than their counterparts under *ϕ*_2_, on each region.

### 5.2 Results

We consider three simulation examples. For each example, we let ***X*** be uniformly sampled from $X=[0,1{]}^{5}$ and $X=[0,1{]}^{10}$ respectively. For simplicity, we assume *A* ⊥ ***X*** and *p*(*A*∣***X***) = 1/*k*, and let *Y* = *μ_A_*(***X***)+*ϵ* where *ϵ* ~ *N*(0, 1/2). In each case, we let the training sample size to be *n* = 500, 1000, 2000, and use a test set with sample size 1000 to evaluate the performance. We compare three methods, namely, the regression-based method, the two-step classification-based method with squared loss, and the one-step classification-based method with the bent hinge loss. For each method, we output both ITR and A-ITR with *c* = 1.2. Finally, we repeat each simulation 100 times and report the averages.

**Figure 4:**
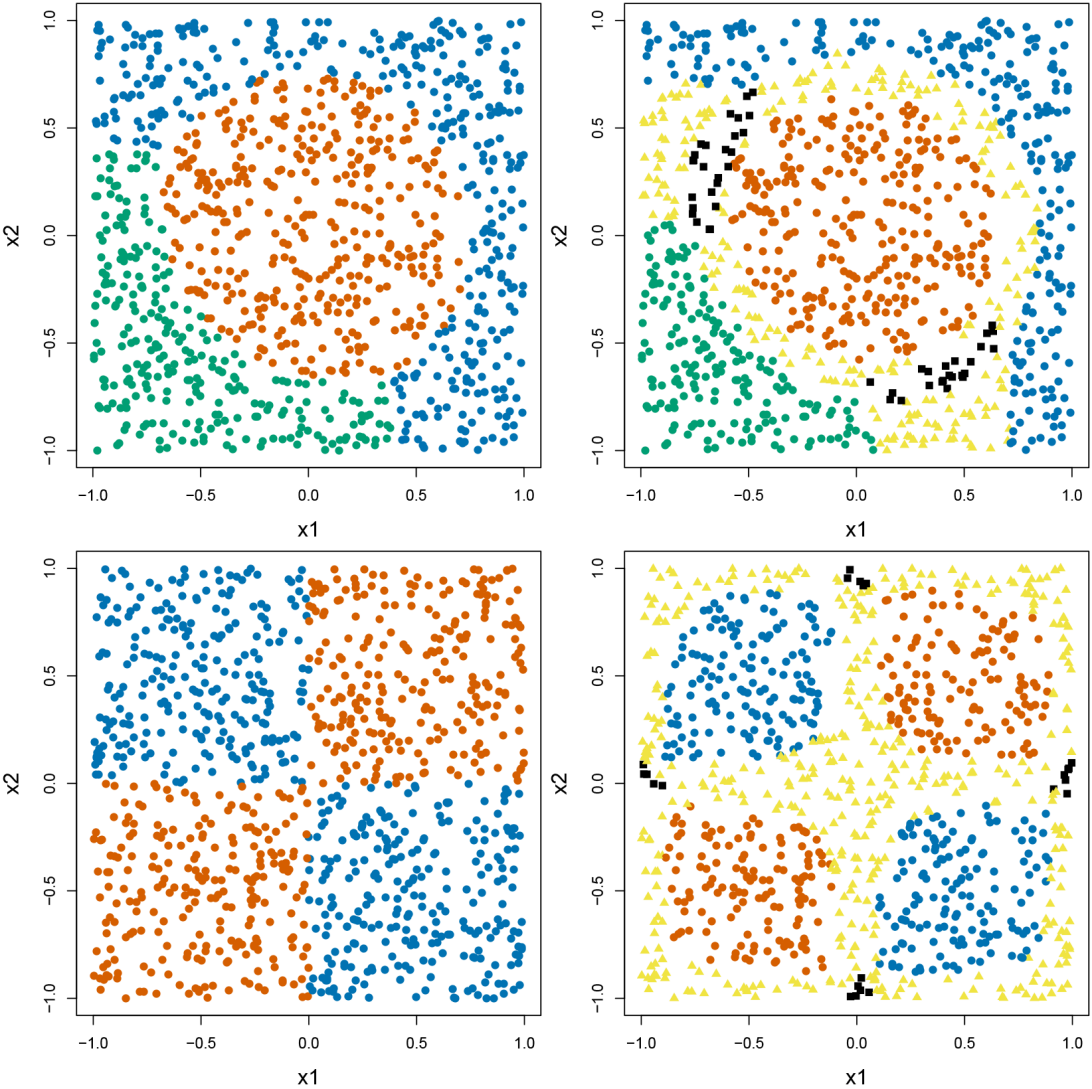
The true ITR and A-ITR for observations in Examples 1 and 2 with recommendation types shown in colors. The orange, blue and green dots indicate that only one single treatment is suggested, which by definition is *R*_1_, while the yellow triangles indicate *R*_2_, and the black squares indicate *R*_3_. The upper panel is Example 1, the lower panel Example 2. The left panel is ITR, and the right panel is A-ITR (*c* = 1.2).

**Example 1:** This is an example with three treatments, where two conditional mean outcome functions are polynomial and the other is linear. Specifically, we have $\mu_{1}(X)=1+3X_{1}^{2}+3X_{2}^{2}$, $\mu_{2}(X)=3-0.5X_{1}^{2}+0.5X_{2}^{2}$, and *μ*_3_(***X***) = 3 + *X*_1_ + *X*_2_. The upper panel in Figure 4 shows the true boundaries for the three treatments. We use polynomial kernel for both the two-step and one-step methods. The tuning parameter *λ* is chosen from 5^−6^ to 5^2^.

**Example 2:** This is an example with four treatments, where all the conditional mean outcome functions are non-linear, *μ_A_*(***X***) = 2 + sign(*A* − 2.5) cos (0.5*π*(*X*_1_ + (−1)^*A*^*X*_2_)). Specifically, treatment 2 and 4 are dominated by treatments 1 and 3 and the optimal ITR should only output either 1 or 3. However, in certain regions treatment 2 and 4 still produce fairly good outcomes which can only be captured by A-ITR. The lower panel in Figure 4 shows the true boundaries. For the two-step method, we report the results using Gaussian kernel. For the one-step method, we report the results with polynomial kernel. The tuning parameter *λ* is chosen from 5^−9^ to 5^−1^.

**Example 3:** This is an example where Assumption 8 is violated. Specifically, $\mu_{1}(X)=\max(2.5,2.3+X_{1}^{2}+X_{2}^{2})$, $\mu_{2}(X)=2.7-2*X_{1}+\text{exp}(X_{3}^{2})-X_{4}^{3}$, and $\mu_{3}(X)=\text{min}(3,3.2-X_{1}^{2}-X_{2}^{2})$. Note that when *c* = 1.2, *p*(*μ*_3_(***X***) = *cμ*_1_(***X***)) > 0 so Assumption 8 is violated. Similar to Example 1, we report the results using polynomial kernel for both the two-step and one-step methods. The tuning parameter *λ* is chosen from 5^−7^ to 1.

Table 1 collects the results of the three examples with *n* = 1000 with dimension *p* = 5 and 10. The results with *n* = 500 and 2000 are provided in the supplementary material. In Table 1, the results of A-ITR are in the form of intervals while the results of ITR are single numbers. We also compute the empirical weighted outcome (“All” column in Table 1) defined in (13) as an indicator for the overall performance for each method.

We note that the performance intervals for A-ITR always cover the expected outcomes of the single-valued ITR. This implies that by applying our proposed A-ITR framework, patients will potentially get a much better outcome as long as they are willing to consider other equally effective options identified by the A-ITR. Even if the patient does not choose the best option within the recommendation set, the worst case is not too bad and the ratio of its outcome to that of the best option is about *c* if the A-ITR is accurately estimated.

We compare different methods by inspecting the length and location of the A-ITR performance interval. Recall that the A-ITR with the shortest interval, the smallest lower limit, and the smallest upper limit on each region is the best A-ITR. However, since *R*_3_ is the region where all treatments are near the optimality, different recommendations are expected to perform similarly. Hence we focus on regions *R*_1_ and *R*_2_ for the purpose of comparison.

From Table 1, we note that the regression-based A-ITR, though has the smallest lower limit in some cases, also has a longer interval in most cases, suggesting that the treatment could either go really well or really badly. This implies that the regression-based A-ITR method tends to include ineffective treatments into the near-optimal set. Part of the reason may be that the regression-based method has not accurately estimated each of the three or four potential outcome functions.

For the classification-based A-ITRs, the lower limits are roughly the same between the one-step method and the two-step method; however, the one-step method has shorter intervals in most cases. This means that the one-step method is better at excluding ineffective treatment options from the recommendation than the two-step method. This is true even when Assumption 8 is violated (Example 3). In addition, the one-step method also has the smallest expected weighted outcome (shown in the “All” column). However, the performance of both the one-step method and the two-step method becomes worse as the sample size decreases (see supplementary material), or the number of covariates increases. This may be due to the inefficiency of using the inverse probability weighting in the OWL framework.

## 6. Real Data Analysis

In this section, we apply our proposed A-ITR framework to a Type 2 diabetes mellitus (T2DM) observational study. The data set contains 1139 patients. Every patient was assigned one out of four diabetes treatments, which are GLP-1 receptor agonists alone, long-acting insulin alone, intermediate-acting insulin alone, and insulin regimens including short-acting insulin. The endpoint is the change of hemoglobin A1c level before and after the treatment, which is denoted by Δ*HbA*1*c*. In practice, if the treatment works, this value is usually negative (meaning that the hemoglobin A1c level decreases). The smaller Δ*HbA*1*c* is, the more effective the treatment is.

We first preprocess the original data set. Among the 19 covariates, we exclude those with a large proportion of missing values and with extremely imbalanced categories. We then impute the rest of them using the predictive mean matching method (Van Buuren, 2018). There are 10 covariates left after the preprocessing: gender, diabetic retinopathy, diabetic neuropathy, age, weight, body mass index (BMI), baseline hemoglobin A1c level, baseline high-density lipoprotein cholesterol (HDL), baseline low-density lipoprotein cholesterol (LDL), and heart disease.

For the outcome variable Δ*HbA*1*c*, we can reduce its variability by subtracting an estimate of its conditional mean E(ΔHbA1c∣x) to make the estimation of $\hat{f}$ more robust (Liu et al., 2018; Zhou et al., 2017). Here we use the ordinary least square regression to estimate E(ΔHbA1c∣x). Denote the estimated mean function fitted by regression as $\hat{m}(x)$, we then observe that $\Delta HbA1c-\hat{m}(x)$ can be positive or negative. We perform an exponential transformation to make it positive, which also justifies the use of ratio *μ_j_*/*μ*_(1)_ to determine the near-optimal recommendation set. Specifically, we let $Y=\text{exp}\;((\Delta HbA1c-\hat{m}(x))∕5)$. If we further assume conditional normality for Δ*HbA*1*c* given ***X*** and treatment *j*, with mean $\nu_{j}(x)\equiv E(\Delta HbA1c\;|\;X=x,A=j)$ and equal variance across treatments, then *Y* ∣ (***x***, *j*) follows a log-normal distribution with mean proportional to exp(*v_j_*(***x***)/5). Then the optimal A-ITR is,

$$\phi^{*}(x)=\left\{j\;|\;\frac{\mu_{j}(x)}{\mu_{\left(1\right)}(x)}=\frac{E\;(Y\;|\;X=x,A=j)}{{\text{min}}_{i}\;E(Y\;|\;X=x,A=i)}=\frac{\text{exp}(\nu_{j}(x)∕5)}{\text{exp}(\nu_{\left(1\right)}(x)∕5)}\leq c\right\}\;\;=\{j\;|\;\nu_{j}(x)-\nu_{\left(1\right)}(x)\leq 5\;\log\;c\}.$$

In this study, we choose the near-optimality constant *c* = 1.2, so that 5 log *c* ≈ 0.9. This implies that the near-optimal recommendation set is constructed by including all treatments with conditional means Δ*HbA*1*c* within 0.9 of the optimal treatment.

We compare the performance of the regression-based method, the two-step method, and the one-step method. For both classification-based methods, we estimate the propensity score *p*(*A*∣***X***) using logistic regression. Each method leads to a single-valued ITR and a set-valued A-ITR. In Table 2, we compare the different recommendations using the 5-fold cross-validated empirical weighted outcome defined in (13).

**Figure 5:**
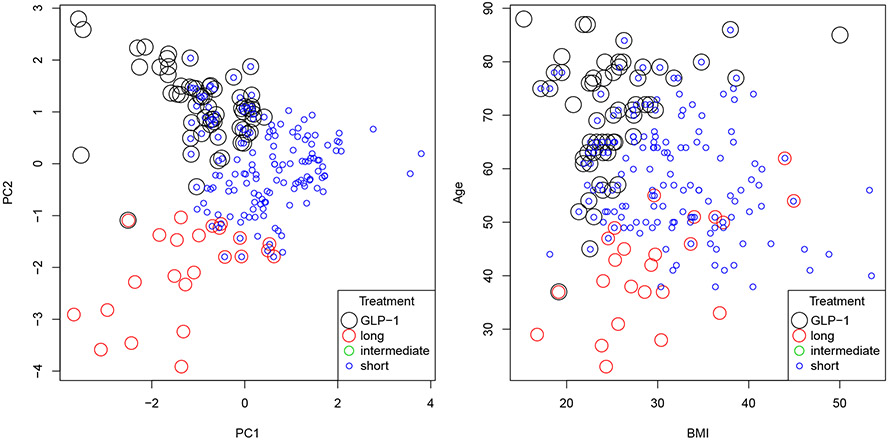
Predicted treatment(s) for patients with recommendations given by different colors. The data set is projected on the first two principal components (left panel), and two particular covariates, age and BMI (right panel). Concentric circles indicate multiple treatments recommended to the same patient.

From Table 2, we observe that the one-step method with Gaussian kernel has the best weighted outcome. To illustrate the resultant A-ITR, we split the data into a training set (70%) and a test set (30%). We fit the training set using the one-step method with Gaussian kernel and then construct the recommendation set for patients in the test set. In our analysis, no patient is recommended to take the intermediate-acting insulin and the majority of patients are recommended to choose between the short-acting insulin and GLP-1. Specifically, 55% of patients are recommended the short-acting insulin only, 8% are recommended GLP-1 only, and 24% are recommended to take either one of the two. For the remaining 13% of patients, they are all recommended to take the long-acting insulin, including 1% who are suggested to take either the long-acting insulin or GLP-1, 5% who are suggested to take either the long-acting insulin or the short-acting insulin, and 7% whose only option is the long-acting insulin. We visualize the predicted treatments in Figure 5.

From Figure 5, we can see that age and BMI are two useful biomarkers in constructing the near-optimal recommendation set. In fact, by comparing the left panel and the right panel of Figure 5, we observe that BMI behaves like the first principal component (PC1) while age behaves like the second principal component (PC2). Figure 5 suggests that for patients without obesity (BMI less than 30), younger patients should take the long-acting insulin while older patients should take GLP-1. The short-acting insulin, on the other hand, serves as an “universal” treatment that many patients can take as an alternative, and is especially effective for overweighted patients.

## 7. Statistical Learning Theory

In this section, we study the convergence rate of the excess *ℓ*-risk in both linear learning and kernel learning settings. We assume the random vector ***Z*** = (***X***, *A*, *Y*) follows a certain distribution *P* that satisfies Assumption 1. Furthermore, we make an additional assumption.

**Assumption 10** *There is a constant C* > 0 *such that* ∣*Y*/*p*(*A*∣***X***)∣ ≤ *C holds. For simplicity, we set C* = 1 *through out this section*.

For ***f*** and ***f′***, two (*k* − 1)-dimensional functions, and *ℓ*, an increasing, convex and Lipchitz loss function, denote

$$e_{\ell }(f,f^{\prime })=E\left[\frac{Y}{p(A|X)}\ell (\langle W_{A},f\rangle )\right]-E\left[\frac{Y}{p(A|X)}\ell (\langle W_{A},f^{\prime }\rangle )\right].$$

We call *e_ℓ_*(***f***, f*) the excess *ℓ*-risk of ***f*** if f* is optimal within a certain function space F.

### 7.1 Linear Learning

We first consider the linear function space, that is, we assume ***f*** = (*f*_1_,…, *f*_*k*−1_)^*T*^ with *f_j_*(***x***) = ***x***^*T*^***β**_j_* for *j* = 1,…, *k* − 1. For simplicity, we assume each covariate is bounded by [0, 1].

**Assumption 11** $X\in X=[0,1{]}^{p}$.

Now consider the following function space,

$$F(p,s)=\{f=(f_{1},\ldots ,f_{k-1}{)}^{T};f_{j}(x)=x^{T}\beta_{j},j=1,\ldots ,k-1,J(f)\leq s\},$$

where $J(f)=\sum_{j=1}^{k-1}\|\beta_{j}{\|}_{2}^{2}=\sum_{j=1}^{k-1}\sum_{l=1}^{p}\beta_{lj}^{2}$. Let $F(p)=\cup_{0\leq s<\infty }F(p,s)$. Define

$$f^{\left(p\right)}=\underset{f\in F(p)}{\text{argmin}}E\left[\frac{Y}{p(A|X)}\ell (\langle W_{A},f(X)\rangle )\right],\;f^{\left(p,s\right)}=\underset{f\in F(p,s)}{\text{argmin}}E\left[\frac{Y}{p(A|X)}\ell (\langle W_{A},f(X)\rangle )\right],\;\;\text{and}\;\;\hat{f}=\underset{f\in F(p,s)}{\text{argmin}}\frac{1}{n}\sum_{i=1}^{n}\frac{y_{i}}{p(a_{i}|x_{i})}\ell (\langle W_{a_{i}},f(x_{i})\rangle ).$$

Theorem 12 gives the convergence rate for the excess *ℓ*-risk $e_{\ell }(\hat{f},f^{\left(p\right)})$, where *p* = *p*_*n*_, *s* = *s*_*n*_ can grow with *n* as *n* → ∞.

**Theorem 12** *Let* $\tau_{n}=(n^{-1}\;\log\;p_{n}{)}^{1∕2}\to 0$ *as n* → ∞. *For linear learning, suppose Assumptions 1, 8, 10, and 11 hold. We have*

$$e_{\ell }(\hat{f},f^{\left(p_{n}\right)})=O\left(\max(c(p_{n}s_{n}{)}^{1∕2}\tau_{n}\;\log\;\tau_{n}^{-1},\delta_{n})\right),$$

*almost surely under P, where δ_n_* = *e_ℓ_*(***f***^(*p_n_*,*s_n_*)^, ***f***^(*p_n_*)^).

In Theorem 12, *δ_n_* stands for the approximation error between the optimal ***f*** in $F(p_{n},s_{n})$ and the optimal ***f*** in $F(p_{n})$. So if *s_n_* → ∞, *δ_n_* converges to 0. On the other hand, the first term $O\left(c(p_{n}s_{n}{)}^{1∕2}\tau_{n}\;\log\;\tau_{n}^{-1}\right)$ is the estimation error between $\hat{f}$ and ***f***^(*p_n_*,*s_n_*)^, and as we increase *s_n_*, it becomes larger. The optimal tuning parameter *s_n_* is then chosen such that $c(p_{n}s_{n}{)}^{1∕2}\tau_{n}\;\log\;\tau_{n}^{-1}∼\delta_{n}$.

In Theorem 12 we may allow *s_n_* → ∞ with an appropriately chosen rate. The reason is that when we include diverging number of covariates, i.e., *p* → ∞, *f*^(*p*)^ can become more complicated, and thus we need a larger *s_n_* to accommodate this change. However, in practice it may not be necessary since the true model usually depends on a finite number of covariates. So we could simplify Theorem 12 if we make the assumption that there is a finite s* such that $f^{\left(p\right)}\in F(p,s^{*})$ for all *p*. For example, suppose $f_{j}^{*}(x)=f_{j}^{\left(p\right)}(x)=\sum_{l=1}^{m}x_{l}\beta_{lj}^{*}$ for *j* = 1,…, *k* − 1 and all *p* = *m*,…, ∞. Then we can choose $s^{*}=\sum_{j=1}^{k-1}\sum_{l=1}^{m}|\beta_{lj}^{*}{|}^{2}$.

**Corollary 13** *Suppose* f* *defined in* (6) *only depends on finite many covariates, and that Assumptions 1, 8, 10 and 11 hold. We have*

$$e_{\ell }(\hat{f},f^{*})=O\left(cp_{n}^{1∕2}\tau_{n}\;\log\;\tau_{n}^{-1}\right)=O\left(c(n^{-1}p_{n}\log\;p_{n}{)}^{1∕2}\log(n(\log\;p_{n}{)}^{-1})\right),$$

*almost surely under P*.

The convergence of excess *ℓ*-risk $e_{\ell }(\hat{f},f^{*})$ in Corollary 13 requires that *p_n_* = *o*(*n*). Particularly, when *p_n_* grows no faster than *n*^1−*r*^, where 0 *< *r* <* 1, it can be verified that the error rate is at an order of no greater than $n^{-r∕2}(\log\;n{)}^{3∕2}$. This result is consistent with most of the classical asymptotic theory that the dimension of covariates should not be greater than the number of observations. Furthermore, we observe that if *p_n_* = *O*(1), then $e_{\ell }(\hat{f},f^{*})=O(n^{-1∕2}\;\log\;n)$, which is almost *O*(*n*^−1/2^).

### 7.2 Kernel Learning

Next we discuss the convergence rate of excess *ℓ*-risk for kernel learning. We denote ***f*** = (*f*_1_,…, *f*_*k*−1_)^*T*^ to be a function in a reproducing kernel Hilbert space (RKHS) *H* with kernel function *K*(·, ·). Then by the RKHS theory, we can write $f_{j}(x)=\sum_{i=1}^{n}K(x_{i},x)\alpha_{ij}+\alpha_{0j}$ for *j* = 1,…, *k* − 1. To develop the theory for the proposed methods, we still need one more assumption.

**Assumption 14** *Suppose H is a separable RKHS equipped with kernel function K*(·, ·). *There exists a positive number B, such that K*(***x***, ***x′***) ≤ *B* *for any* ***x***, $x^{\prime }\in X$.

Assumption 14 states that the RKHS is separable and the kernel function is bounded. This is true for many commonly used kernel functions. For example, for the Gaussian kernel, we may take *B* = 1. We define the function space as

$$F(n,s)=\{f=(f_{1},\ldots ,f_{k-1}{)}^{T}\;|\;f_{j}(x)=\sum_{i=1}^{n}K(x_{i},x)\alpha_{ij}+\alpha_{0j},j=1,\ldots ,k-1,J(f)\leq s\},$$

where $J(f)=\sum_{j=1}^{k-1}\alpha_{j}^{T}K\alpha_{j}+\sum_{j=1}^{k-1}\alpha_{0j}^{2}$ and **K** is the gram matrix. Recall we have included intercepts in the penalty for simplicity. Let $F(\infty )={\text{lim}}_{n\to \infty }\cup_{0\leq s<\infty }F(n,s)$, and define

$$f^{\left(\infty \right)}=\underset{f\in F(\infty )}{\text{argmin}}E\left[\frac{Y}{p(A|X)}\ell (\langle W_{A},f(X)\rangle )\right],\;f^{\left(n,s\right)}=\underset{f\in F(n,s)}{\text{argmin}}E\left[\frac{Y}{p(A|X)}\ell (\langle W_{A},f(X)\rangle )\right],\;\;\text{and}\;\;\hat{f}=\underset{f\in F(n,s)}{\text{argmin}}\frac{1}{n}\sum_{i=1}^{n}\frac{y_{i}}{p(a_{i}|x_{i})}\ell (\langle W_{a_{i}},f(x_{i})\rangle ),$$

The following theorem gives the convergence rate of $e_{\ell }(\hat{f},f^{\left(\infty \right)})$ when *s* = *s_n_* grows with *n*.

**Theorem 15** *For RKHS learning, suppose Assumptions 1, 8, 10 and 14 hold. We have*

$$e_{\ell }(\hat{f},f^{\left(\infty \right)})=O\left(\max(cB(s_{n}∕n{)}^{1∕2}\;\log\;n,\delta_{n})\right),$$

*almost surely under P, where δ_n_* = *e_ℓ_*(***f***^(*n*,*s_n_*)^, ***f***^(∞)^).

Similar to the linear case, there is a trade-off between the approximation error *δ_n_* and the estimation error $O\left(cB(s_{n}∕n{)}^{1∕2}\;\log\;n\right)$ in Theorem 15, and the optimal tuning parameter *s_n_* is determined roughly when $cB(s_{n}∕n{)}^{1∕2}\;\log\;n∼\delta_{n}$.

Compared to Theorem 12, the excess *ℓ*-risk for RKHS learning seems to yield a faster rate. However, this is not always truly the case due to Assumption 14, which requires a bounded kernel function, and implies a restriction on the number of covariates *p*. For example, for linear kernel we have *K*(***x***, ***x′***) = ***x***^*T*^***x′*** ≤ *p* under Assumption 11. For Assumption 14 to be true, we have to let *p* = *O*(1). In this case both convergence rates are $e_{\ell }(\hat{f},f^{\left(p\right)})=O((s_{n}∕n{)}^{1∕2}\;\log\;n)$; that of the kernel learning is no faster than that of the linear learning. In general, to obtain a faster rate than that of the linear learning, we need a kernel function that does not increase in *p*, such as the Gaussian kernel.

Note that the approximation error *δ_n_* converges to 0 as *n* increases, and both the convergence rate of *δ_n_* and that of the resulting $e_{\ell }(\hat{f},f^{*})$ depend on the choice of the kernel. To illustrate the magnitude of *δ_n_* and its impact on the excess risk, consider a binary example where *X* ~ Unif(0, 1) and f*(*x*) = (1 + *x*)^2^. With the polynomial kernel of degree 2 we have *f*^(∞)^ = f* and *B* = max_*x,x′*_(1 + *x,x′*)^2^ = 2. Given a training set {*x*_1_,…, *x_n_*}, let *x*_(*n*)_ be the largest order statistic and define *f*_(*n*)_(*x*) = (1 + *xx*_(*n*)_)^2^. It can be shown that for any *s_n_* ≥ 1, $s_{n}\geq 1,\;f_{\left(n\right)}\subseteq F(n,s_{n})$ thus *δ_n_* = *e_ℓ_*(*f*^(*n*,*s_n_*)^, *f*^(∞)^) ≤ *e_ℓ_*(*f*_(*n*)_, $f^{\left(\infty \right)})\leq cE\|\;f_{\left(n\right)}-f^{\left(\infty \right)}{\|}_{2}$. Note that the difference between *f*_(*n*)_ and *f*^(∞)^ is maximized at 1, so $\delta_{n}\leq cE|f_{\left(n\right)}(1)-f^{\left(\infty \right)}(1)|=cE\left(3-2x_{\left(n\right)}-x_{\left(n\right)}^{2}\right)$. Because the density function of *x*_(*n*)_ is *nx*^*n*−1^**1**_(0,1_), we have $\delta_{n}\leq c\int_{0}^{1}(3-2x-x^{2})nx^{n-1}dx=\frac{2c(2n+3)}{\left(n+1)(n+2\right)}$. Hence in this example, the order of *δ_n_* is at most *O*(*n*^−1^), thus $e_{\ell }(\hat{f},f^{*})=e_{\ell }(\hat{f},f^{\left(\infty \right)})=O(n^{-1∕2}\;\log\;n)$.

## 8. Conclusion and Discussions

In this work, we propose a new individualized treatment recommendation framework, named A-ITR, that has the capacity to recommend to patients near-optimal treatment options in terms of their clinical outcomes. By adopting the A-ITR, patients have the opportunity to choose the treatment options tailored for their different financial situations, personal preferences, and lifestyle choices. To estimate the optimal A-ITR, we proposed two classification-based methods based on the OWL framework. We also provide a new evaluation criterion suitable for A-ITRs, namely the weighted expected outcome, defined in (12). The simulation study shows the usefulness of this new criterion in parameter tuning and model selection.

The proposed methods may be subject to misuse when caution is not exercised with regard to the choice of the near-optimality constant *c*. Typically, *c* is chosen based on the physicians’ experience on what defines near optimality for a particular outcome. When possible, the choice could be made more objective by using a measure of the variability of the outcomes for the top treatments. Lastly, recommendations from a variety of *c* values could be presented to the patients for a final selection, as long as any possible sacrifice of the outcome can be clearly explained to the patients.

There are several possible directions for future works. Firstly, the current A-ITR estimation is based on the OWL framework, which may be sensitive to the estimated propensity score. In particular, the OWL estimator is known to suffer a large variance in practice (Zhou et al., 2017). To address this issue, one may consider applying the recently proposed augmented OWL framework (Zhao et al., 2019; Huang et al., 2019) to improve the finite sample performance. These methods typically have a double robustness property so that the efficiency of the estimator can be further improved. Secondly, when applying the A-ITR framework in practice, it may be desirable to adjust *c* for different needs or preferences. A natural generalization of the method is to incorporate the patients’ preferences on multiple outcomes into the framework. For example, we can consider A-ITRs with an additional competing outcome as a secondary endpoint (Laber et al., 2014), or A-ITR with additional safety endpoints formulated as constraints (Wang et al., 2018). Thirdly, besides the kernel method, we can consider other learning algorithms to estimate $\hat{f}$ within the A-ITR framework. Finally, the current method assumes the outcome *Y* is continuous. We can consider nontrivial extensions to other types of outcome such as count outcomes, survival outcomes (Zhao et al., 2014; Qi et al., 2019) or dichotomous outcomes (Qi et al., 2019; Klausch et al., 2018).

## Figures and Tables

**Table 1:** Results of the simulation studies with *n* = 1000. In each region, the expected outcome for ITR and the outcome interval for A-ITR (*c* = 1.2) are reported. The empirical weighted outcome defined in (13) is shown in the “All” column. Each number is averaged over 100 replications. In each case, the best performing method is marked in bold.

Example 1		*p* = 5				*p* = 10			
		*R*_1_ (70.02%)	*R*_2_ (24.71%)	*R*_3_ (5.27%)	All	*R*_1_ (70.02%)	*R*_2_ (24.71%)	*R*_3_ (5.27%)	All
Reg.	ITR	2.49	2.55	2.61	2.51	2.49	2.55	2.61	2.51
	A-ITR	(1.99, 3.07)	(2.42, 3.00)	(2.47, 2.95)	2.37	(2.03, 3.00)	(2.42, 2.97)	(2.48, 2.94)	**2.37**
2-step	ITR	2.34	2.70	2.69	2.45	2.74	2.86	2.71	2.77
	A-ITR	(2.17, 2.66)	(2.58, 2.87)	(2.6, 2.79)	2.41	(2.47, 3.11)	(2.69, 3.07)	(2.64, 2.79)	2.69
1-step	ITR	2.15	2.62	2.68	2.30	2.43	2.72	2.70	2.51
	A-ITR	(2.04, 2.36)	(2.53, 2.73)	(2.61, 2.75)	**2.26**	(2.21, 2.74)	(2.6, 2.88)	(2.62, 2.78)	2.45
Bayes	ITR	1.92	2.44	2.55	2.08				
	A-ITR	1.92	(2.38, 2.75)	(2.45, 2.99)	2.05				
Example 2		*p* = 5				*p* = 10			
		*R*_1_ (56.78%)	*R*_2_ (41:84%)	*R*_3_ (1.38%)	All	*R*_1_ (56.78%)	*R*_2_ (41.84%)	*R*_3_ (1.38%)	All
Reg.	ITR	1.56	1.61	1.93	1.59	1.57	1.62	1.94	1.59
	A-ITR	(1.15, 1.99)	(1.24, 2.02)	(1.80, 2.07)	1.46	(1.19, 1.95)	(1.27, 1.98)	(1.81, 2.08)	1.47
2-step	ITR	1.29	1.32	1.93	1.32	1.47	1.45	1.95	1.47
	A-ITR	(1.18, 1.52)	(1.23, 1.50)	(1.85, 2.04)	1.30	(1.29, 1.79)	(1.31, 1.75)	(1.86, 2.06)	1.46
1-step	ITR	1.29	1.32	1.93	1.31	1.42	1.42	1.95	1.43
	A-ITR	(1.18, 1.48)	(1.24, 1.44)	(1.85, 2.00)	**1.28**	(1.25, 1.67)	(1.29, 1.62)	(1.86, 2.03)	**1.39**
Bayes	ITR	1.13	1.25	1.87	1.19				
	A-ITR	1.13	(1.15, 1.45)	(1.71, 2.28)	1.16				
Example 3		*p* = 5				*p* = 10			
		*R*_1_ (38.63%)	*R*_2_ (54.13%)	*R*_3_ (7.24%)	All	*R*_1_ (38.63%)	*R*_2_ (54.13%)	*R*_3_ (7.24%)	All
Reg.	ITR	2.17	2.74	2.78	2.52	2.17	2.75	2.76	2.53
	A-ITR	(2.12, 2.92)	(2.54, 2.89)	(2.58, 2.92)	2.46	(2.12, 2.87)	(2.57, 2.89)	(2.59, 2.91)	**2.47**
2-step	ITR	2.37	2.72	2.75	2.59	2.70	2.87	2.76	2.80
	A-ITR	(2.21, 2.87)	(2.53, 3.12)	(2.55, 2.95)	2.51	(2.42, 3.23)	(2.66, 3.24)	(2.62, 2.90)	2.71
1-step	ITR	2.31	2.67	2.73	2.54	2.46	2.74	2.74	2.63
	A-ITR	(2.18, 2.67)	(2.54, 2.84)	(2.62, 2.83)	**2.45**	(2.24, 2.93)	(2.58, 2.95)	(2.64, 2.86)	2.54
Bayes	ITR	2.11	2.51	2.60	2.36				
	A-ITR	2.11	(2.46, 2.88)	(2.50, 3.04)	2.30				

**Table 2:** The mean 5-fold cross validated weighted outcome and its standard error (in the parenthesis) over 100 replications for T2DM data. The method that yields the best result is marked in bold.

	ITR	A-ITR
Regression	1.071 (0.010)	0.988 (0.006)
Two-step: Linear	0.995 (0.007)	0.975 (0.006)
Two-step: Gaussian	0.959 (0.006)	0.947 (0.005)
One-step: Linear	1.150 (0.008)	1.033 (0.006)
One-step: Gaussian	**0.939** (0.006)	**0.935** (0.007)

## Footnotes

*. Although class labels are traditionally denoted as *Y* in the classification literature, the class labels are analogous to the treatment options in the ITR problem. Hence we denote the class label as *A* here.
